# Constructing knowledge graphs and their biomedical applications

**DOI:** 10.1016/j.csbj.2020.05.017

**Published:** 2020-06-02

**Authors:** David N. Nicholson, Casey S. Greene

**Affiliations:** a Department of Systems Pharmacology and Translational Therapeutics, University of Pennsylvania, United States; b Department of Systems Pharmacology and Translational Therapeutics, Perelman School of Medicine, University of Pennsylvania, Childhood Cancer Data Lab, Alex’s Lemonade Stand Foundation, United States

**Keywords:** knowledge graphs, Network embeddings, Text mining, Natural language processing, Machine learning, Lterature review

## Abstract

Knowledge graphs can support many biomedical applications. These graphs represent biomedical concepts and relationships in the form of nodes and edges. In this review, we discuss how these graphs are constructed and applied with a particular focus on how machine learning approaches are changing these processes. Biomedical knowledge graphs have often been constructed by integrating databases that were populated by experts via manual curation, but we are now seeing a more robust use of automated systems. A number of techniques are used to represent knowledge graphs, but often machine learning methods are used to construct a low-dimensional representation that can support many different applications. This representation is designed to preserve a knowledge graph’s local and/or global structure. Additional machine learning methods can be applied to this representation to make predictions within genomic, pharmaceutical, and clinical domains. We frame our discussion first around knowledge graph construction and then around unifying representational learning techniques and unifying applications. Advances in machine learning for biomedicine are creating new opportunities across many domains, and we note potential avenues for future work with knowledge graphs that appear particularly promising.

## 1 Introduction

Graphs are practical resources for many real-world applications. They have been used in social network mining to classify nodes [1] and create recommendation systems [2]. They have also been used in natural language processing to interpret simple questions and use relational information to provide answers [3], [4]. In a biomedical setting, graphs have been used to prioritize genes relevant to disease [5], [6], [7], [8], perform drug repurposing [9] and identify drug-target interactions [10].

**Fig. 1:**
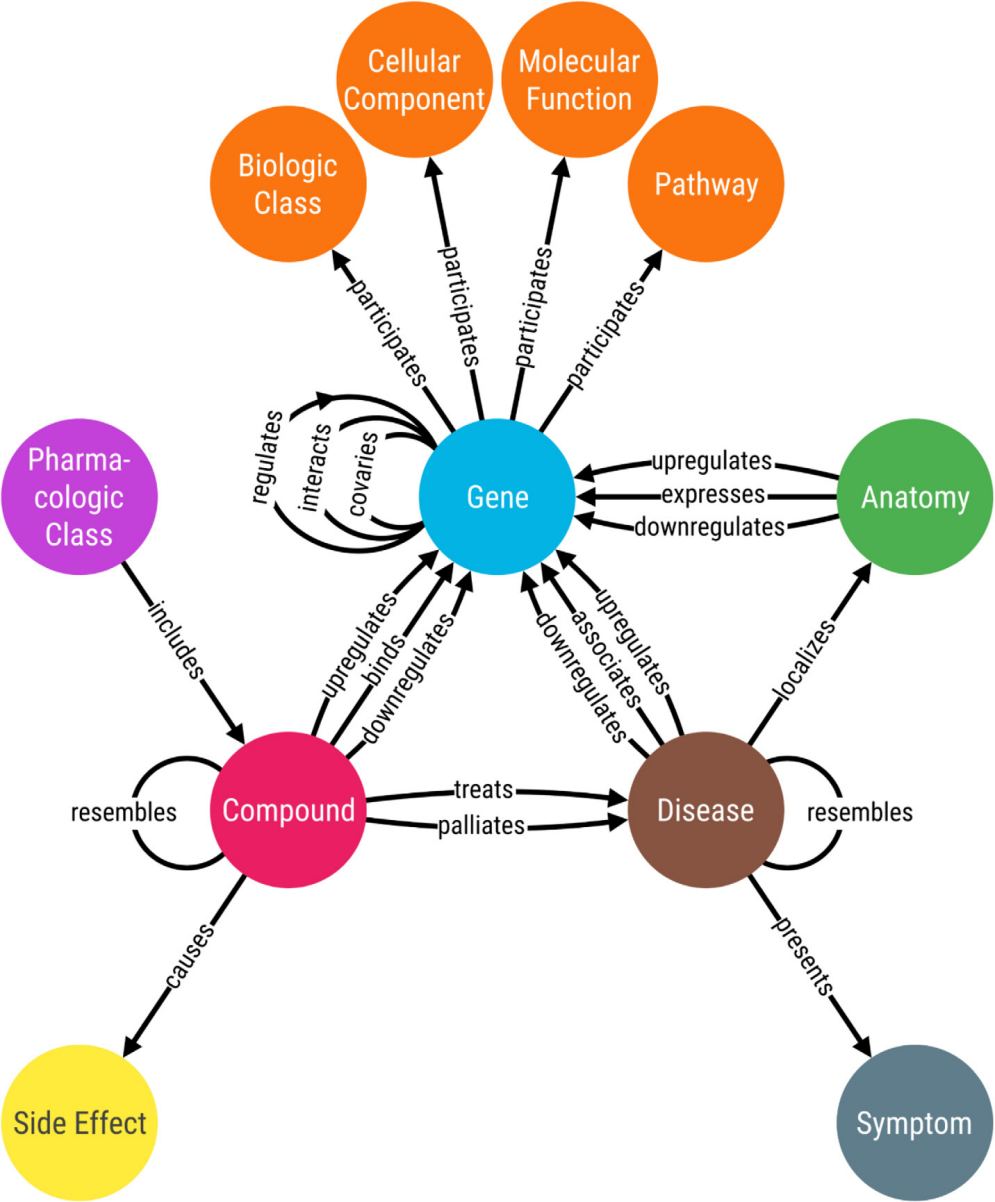
The metagraph (i.e., schema) of the knowledge graph used in the Rephetio project [9]. The authors of this project refer to their resource as a heterogenous network (i.e., hetnet), and this network meets our definition of a knowledge graph. This resource depicts pharmacological and biomedical information in the form of nodes and edges. The nodes (circles) represent entities and edges (lines) represent relationships that are shared between two entities. The majority of edges in this metagraph are depicted as unidirectional, but some relationships can be considered bidirectional.

Within a biomedical setting, some graphs can be considered knowledge graphs; although, precisely defining a knowledge graph is difficult because there are multiple conflicting definitions [11]. For this review, we define a biomedical knowledge graph as the following: a resource that integrates one or more expert-derived sources of information into a graph where nodes represent biomedical entities and edges represent relationships between two entities. This definition is consistent with other definitions found in the literature [12], [13], [14], [15], [16], [17], [18]. Often relationships are considered unidirectional (e.g., a compound treats a disease, but a disease cannot treat a compound); however, there are cases where relationships can be considered bidirectional (e.g., a compound resembles another compound, or a gene interacts with another gene). A subset of graphs that meet our definition of a knowledge graph would be unsuitable for applications such as symbolic reasoning [19]; however, we chose a more liberal definition because it has been demonstrated that these broadly defined graphs have numerous uses throughout the literature. For example, Hetionet (Fig. 1) [9] would be considered a biomedical knowledge graph by this definition, and it has been used to identify drug repurposing opportunities [9]. We do not consider databases like DISEASES [20] and DrugBank [21] to be knowledge graphs. Although these resources contain essential information, they do not represent their data in the form of a graph.

Biomedical knowledge graphs are often constructed from manually curated databases [10], [22], [23], [24], [9]. These databases provide previously established information that can be incorporated into a graph. For example, a graph using DISEASES [20] as a resource would have genes and diseases as nodes, while edges added between nodes would represent an association between a gene and a disease. This example shows a single type of relationship; however, there are graphs that use databases with multiple relationships [25], [9]. In addition to manual curation, other approaches have used natural language processing techniques to construct knowledge graphs [26], [27]. One example used a text mining system to extract sentences that illustrate a protein’s interaction with another protein [28]. Once identified, these sentences can be incorporated as evidence to establish an edge in a knowledge graph.

In this review we describe various approaches for constructing and applying knowledge graphs in a biomedical setting. We discuss the pros and cons of constructing a knowledge graph via manually curated databases and via text mining systems. We also compare assorted approaches for applying knowledge graphs to solve biomedical problems. Lastly, we conclude on the practicality of knowledge graphs and point out future applications that have yet to be explored.

## 2 Building biomedical knowledge graphs

Knowledge graphs can be constructed in many ways using resources such as pre-existing databases or text. Usually, knowledge graphs are constructed using pre-existing databases. These databases are constructed by domain experts using approaches ranging from manual curation to automated techniques, such as text mining. Manual curation is a time-consuming process that requires domain experts to read papers and annotate sentences that assert a relationship. Automated approaches rely on machine learning or natural language processing techniques to rapidly detect sentences of interest. We categorize these automated approaches into the following groups: rule-based extraction, unsupervised machine learning, and supervised machine learning and discuss examples of each type of approach while synthesizing their strengths and weaknesses.

### 2.1 Constructing databases and manual curation

Database construction dates back all the way to 1956 when the first database contained a protein sequence of the insulin molecule [29]. The process of database construction involves gathering relevant text such as journal articles, abstracts, or web-based text and having curators read the gathered text to detect sentences that implicate a relationship (i.e., relationship extraction). Notable databases constructed by this process can be in found in Table 1. An example database, COSMIC [30] was constructed by a group of domain experts scanning the literature for key cancer related genes. This database contained approximately 35 M entries in 2016 [30] and by 2018 had grown to 45 M entries [31]. Studies have shown that databases constructed in this fashion contain relatively precise data but the recall is low [32], [33], [34], [35], [36], [37], [38]. Low recall happens because the publication rate is too high for curators to keep up [39]. This bottleneck highlights a critical need for future approaches to scale fast enough to compete with the increasing publication rate.

**Table 1:** A table of databases that used a form of manual curation to populate entries. Reported number of entities and relationships are relative to the time of publication.

Database [Reference]	Short Description	Number of Entries	Entity Types	Relationship Types	Method of Population
BioGrid [52]	A database for major model organisms. It contains genetic and proteomic information.	572,084	Genes, Proteins	Protein-Protein interactions	Semi-automatic methods
Comparative Toxicogenomics Database [53]	A database that contains manually curated chemical-gene-disease interactions and relationships.	2,429,689	Chemicals (Drugs), Genes, Diseases	Drug-Genes, Drug-Disease, Disease-Gene mappings	Manual curation and Automated systems
Comprehensive Antibiotic Resistance Database [54]	Manually curated database that contains information about the molecular basis of antimicrobial resistance.	174,443	Drugs, Genes, Variants	Drug-Gene, Drug-Variant mappings	Manual curation
COSMIC [30]	A database that contains high resolution human cancer genetic information.	35,946,704	Genes, Variants, Tumor Types	Gene-Variant Mappings	Manual Curation
Entrez-Gene [55]	NCBI’s Gene annotation database that contains information pertaining to genes, gene’s organism source, phenotypes etc.	7,883,114	Genes, Species and Phenotypes	Gene-Phenotypes and Genes-Species mappings	Semi-automated curation
OMIM [56]	A database that contains phenotype and genotype information	25,153	Genes, Phenotypes	Gene-Phenotype mappings	Manual Curation
PharmGKB [57]	A database that contains genetic, phenotypic, and clinical information related to pharmacogenomic studies.	43,112	Drugs, Genes, Phenotypes, Variants, Pathways	Gene-Phenotypes, Pathway-Drugs, Gene-Variants, Gene-Pathways	Manual Curation and Automated Methods
UniProt [58]	A protein–protein interaction database that contains proteomic information.	560,823	Proteins, Protein sequences	Protein-Protein interactions	Manual and Automated Curation

Semi-automatic methods are a way to accelerate the curation process [36], [40], [41], [42], [43], [44], [45]. The first step of these methods is to use an automated system to initially extract sentences from text. This process removes irrelevant sentences, which dramatically decreases the amount of text that curators must sift through. Following the pre-filtering step, curators then approve or reject the remaining sentences. This approach saved curators an average of 2–2.8 h compared to manual efforts [40], [46]. Despite automated systems excelling in identifying sentences for commonly occurring relationships, they tend to miss lesser-known relationships [40]. These systems also have a hard time parsing ambiguous sentences that naturally occur in text, which makes correcting them a challenging task [40]. Given these caveats, future approaches should look into using techniques that simplify sentences to solve the ambiguity issue [47], [48].

Despite the negatives of manual curation, it is still an essential process for extracting relationships from text. This process can be used to generate gold standard datasets that automated systems use for validation [49], [50] and can be used during the training process of these systems (i.e., active learning) [51]. It is important to remember that manual curation alone is precise but results in low recall rates [38]. Future databases should consider initially relying on automated methods to obtain sentences at an acceptable recall level, then incorporate manual curation as a way to fix or remove irrelevant results.

### 2.2 Text mining for relationship extraction

#### 2.2.1 Rule-based relationship extraction

**Fig. 2:**
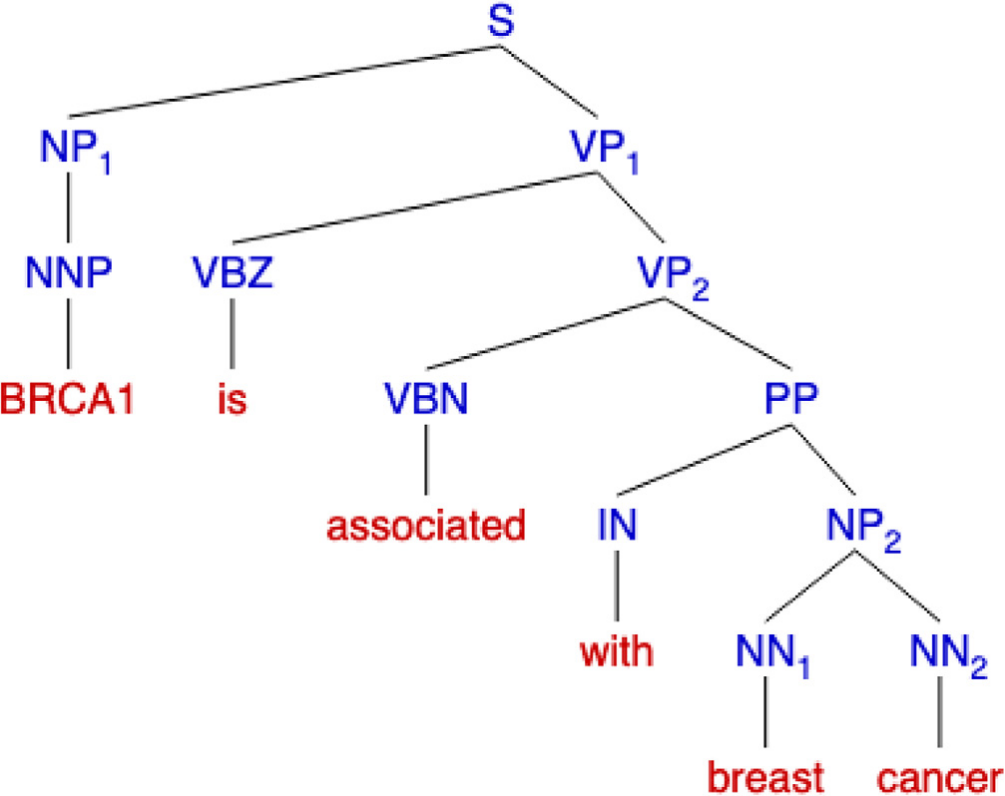
A visualization of a constituency parse tree using the following sentence: “BRCA1 is associated with breast cancer” [73]. This type of tree has the root start at the beginning of the sentence. Each word is grouped into subphrases depending on its correlating part of speech tag. For example, the word “associated” is a past participle verb (VBN) that belongs to the verb phrase (VP) subgroup.

**Fig. 3:**
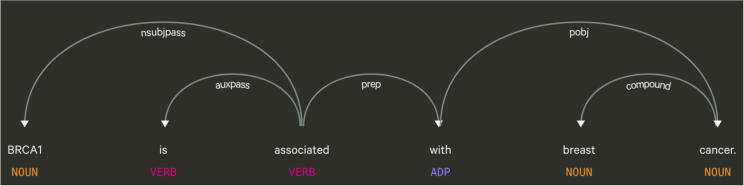
A visualization of a dependency parse tree using the following sentence: “BRCA1 is associated with breast cancer” [74]. For these types of trees, the root begins with the main verb of the sentence. Each arrow represents the dependency shared between two words. For example, the dependency between BRCA1 and associated is nsubjpass, which stands for passive nominal subject. This means that “BRCA1” is the subject of the sentence and it is being referred to by the word “associated”.

Rule-based extraction consists of identifying essential keywords and grammatical patterns to detect relationships of interest. Keywords are established via expert knowledge or through the use of pre-existing ontologies, while grammatical patterns are constructed via experts curating parse trees. Parse trees are tree data structures that depict a sentence’s grammatical structure and come in two forms: a constituency parse tree (Fig. 2) and a dependency parse tree (Fig. 3). Both trees use part of speech tags, labels that dictate the grammatical role of a word such as noun, verb, adjective, etc., for construction, but represent the information in two different forms. Constituency parse trees break a sentence into subphrases (Fig. 2) while dependency path trees analyze the grammatical structure of a sentence (Fig. 3). Many text mining approaches [59], [60], [61] use such trees to generate features for machine learning algorithms and these approaches are discussed in later sections. In this section we focus on approaches that use rule-based extraction as a primary strategy to detect sentences that allude to a relationship.

Grammatical patterns can simplify sentences for easy extraction [48], [62]. Jonnalagadda et al. used a set of grammar rules inspired by constituency trees to reshape complex sentences with simpler versions [48] and these simplified versions were manually curated to determine the presence of a relationship. By simplifying sentences, this approach achieved high recall, but had low precision [48]. Other approaches used simplification techniques to make extraction easier [63], [64], [65], [66]. Tudor et al. simplified sentences to detect protein phosphorylation events [65]. Their sentence simplifier broke complex sentences that contain multiple protein events into smaller sentences that contain only one distinct event. By breaking these sentences down the authors were able to increase their recall; however, sentences that contained ambiguous directionality or multiple phosphorylation events were too complex for the simplifier. As a consequence, the simplifier missed some relevant sentences [65]. These errors highlight a crucial need for future algorithms to be generalizable enough to handle various forms of complex sentences.

Pattern matching is a fundamental approach used to detect relationship asserting sentences. These patterns can consist of phrases from constituency trees, a set of keywords or some combination of both [36], [67], [68], [69], [70], [71]. Xu et al. designed a pattern matcher system to detect sentences in PubMed abstracts that indicate drug-disease treatments [70]. This system matched drug-disease pairs from ClinicalTrials.gov to drug-disease pairs mentioned in abstracts. This matching process aided the authors in identifying sentences that can be used to create simple patterns, such as “Drug in the treatment of Disease” [70], to match other sentences in a wide variety of abstracts. The authors hand curated two datasets for evaluation and achieved a high precision score of 0.904 and a low recall score of 0.131 [70]. This low recall score was based on constructed patterns being too specific to detect infrequent drug pairs. Besides constituency trees, some approaches used dependency trees to construct patterns [59], [72]. Depending upon the nature of the algorithm and text, dependency trees could be more appropriate than constituency trees and vice versa. The performance difference between the two trees remains as an open question for future exploration.

Rule-based methods provide a basis for many relationship extraction systems. Approaches in this category range from simplifying sentences for easy extraction to identifying sentences based on matched key phrases or grammatical patterns. Both require a significant amount of manual effort and expert knowledge to perform well. A future direction is to develop ways to automate the construction of these hand-crafted patterns, which would accelerate the process of creating these rule-based systems.

#### 2.2.2 Extracting relationships without labels

Unsupervised extractors draw inferences from textual data without the use of annotated labels. These methods involve some form of clustering or statistical calculations. In this section we focus on methods that use unsupervised learning to extract relationships from text.

An unsupervised extractor can exploit the fact that two entities may appear together in text. This event is referred to as co-occurrence and studies that use this phenomenon can be found in Table 2. Two databases DISEASES [20] and STRING [75] were populated using a co-occurrence scoring method on PubMed abstracts, which measured the frequency of co-mention pairs within individual sentences as well as the abstracts themselves. This technique assumes that each individual co-occurring pair is independent from one another. Under this assumption mention pairs that occur more than expected were presumed to implicate the presence of an association or interaction. This approach identified 543,405 disease gene associations [20] and 792,730 high confidence protein–protein interactions [75] but is limited to only PubMed abstracts.

**Table 2:** Table of approaches that mainly use a form of co-occurrence.

Study	Relationship of Interest
CoCoScore [79]	Protein-Protein Interactions, Disease-Gene and Tissue-Gene Associations
Rastegar-Mojarad et al. [80]	Drug Disease Treatments
CoPub Discovery [81]	Drug, Gene and Disease interactions
Westergaard et al. [76]	Protein-Protein Interactions
DISEASES [20]	Disease-Gene associations
STRING [82]	Protein-Protein Interactions
Singhal et al. [83]	Genotype-Phenotype Relationships

Full text articles are able to dramatically enhance relationship detection [76], [77]. Westergaard et al. used a co-occurrence approach, similar to DISEASES [20] and STRING [75], to mine full articles for protein–protein interactions and other protein related information [76]. The authors discovered that full text provided better prediction power than using abstracts alone, which suggests that future text mining approaches should consider using full text to increase detection power.

Unsupervised extractors often treat different biomedical relationships as multiple isolated problems. An alternative to this perspective is to capture all different types at once. Clustering is an approach that performs simultaneous extraction. Percha et al. used a biclustering algorithm on generated dependency parse trees to group sentences within PubMed abstracts [78]. Each cluster was manually curated to determine which relationship each group represented. This approach captured 4,451,661 dependency paths for 36 different groups [78]. Despite the success, this approach suffered from technical issues such as dependency tree parsing errors. These errors resulted in some sentences not being captured by the clustering algorithm [78]. Future clustering approaches should consider simplifying sentences to prevent this type of issue.

Overall unsupervised methods provide a means to rapidly extract relationship asserting sentences without the need of annotated text. Approaches in this category range from calculating co-occurrence scores to clustering sentences and provide a generalizable framework that can be used on large repositories of text. Full text has already been shown to meaningfully improve the performance of methods that aim to infer relationships using cooccurrences [76], and we should expect similar benefits for machine learning approaches. Furthermore, we expect that simplifying sentences would improve unsupervised methods and should be considered as an initial preprocessing step.

#### 2.2.3 Supervised relationship extraction

Supervised extractors use labeled sentences to construct generalized patterns that bisect positive examples (sentences that allude to a relationship) from negative ones (sentences that do not allude to a relationship). Most of these approaches have flourished due to pre-labelled publicly available datasets (Table 3). These datasets were constructed by curators for shared open tasks [84], [85] or as a means to provide the scientific community with a gold standard [85], [86], [87]. Approaches that use these available datasets range from using linear classifiers such as support vector machines (SVMs) to non-linear classifiers such as deep learning techniques. The rest of this section discusses approaches that use supervised extractors to detect relationship asserting sentences.

**Table 3:** A set of publicly available datasets for supervised text mining.

Dataset	Type of Sentences
AIMed [50]	Protein-Protein Interactions
BioInfer [123]	Protein-Protein Interactions
LLL [124]	Protein-Protein Interactions
IEPA [125]	Protein-Protein Interactions
HPRD5 [86]	Protein-Protein Interactions
EU-ADR [49]	Disease-Gene Associations
BeFree [90]	Disease-Gene Associations
CoMAGC [87]	Disease-Gene Associations
CRAFT [126]	Disease-Gene Associations
Biocreative V CDR [85]	Compound induces Disease
Biocreative IV ChemProt [84]	Compound-Gene Bindings

Some supervised extractors involve the mapping of textual input into a high dimensional space. SVMs are a type of classifier that can accomplish this task with a mapping function called a kernel [61], [88]. These kernels take information such as a sentence’s dependency tree [59], [60], part of speech tags [61] or even word counts [88] and map them onto a dense feature space. Within this space, these methods construct a hyperplane that separates sentences in the positive class (illustrates a relationship) from the negative class (does not illustrate a relationship). Kernels can be manually constructed or selected to cater to the relationship of interest [60], [61], [88], [88]. Determining the correct kernel is a nontrivial task that requires expert knowledge to be successful. In addition to single kernel methods, a recent study used an ensemble of SVMs to extract disease-gene associations [89]. This ensemble outperformed notable disease-gene association extractors [72], [90] in terms of precision, recall and F1 score. Overall, SVMs have been shown to be beneficial in terms of relationship mining; however, major focus has shifted to utilizing deep learning techniques which can perform non-linear mappings of high dimensional data.

Deep learning is an increasingly popular class of techniques that can construct their own features within a high dimensional space [91], [92]. These methods use different forms of neural networks, such as recurrent or convolutional neural networks, to perform classification.

Recurrent neural networks (RNN) are designed for sequential analysis and use a repeatedly updating hidden state to make predictions. An example of a recurrent neural network is a long short-term memory (LSTM) network [93]. Cocos et al. [94] used a LSTM to extract drug side effects from de-identified twitter posts, while Yadav et al. [95] used an LSTM to extract protein–protein interactions. Others have also embraced LSTMs to perform relationship extraction [94], [96], [97], [98], [99]. Despite the success of these networks, training can be difficult as these networks are highly susceptible to vanishing and exploding gradients [100], [101]. One proposed solution to this problem is to clip the gradients while the neural network trains [102]. Besides the gradient problem, these approaches only peak in performance when the datasets reach at least tens of thousands of data points [103].

Convolutional neural networks (CNNs), which are widely applied for image analysis, use multiple kernel filters to capture small subsets of an overall image [92]. In the context of text mining an image is replaced with words within a sentence mapped to dense vectors (i.e., word embeddings) [104], [105]. Peng et al. used a CNN to extract sentences that mentioned protein–protein interactions [106] and Zhou et al. used a CNN to extract chemical-disease relations [107]. Others have used CNNs and variants of CNNs to extract relationships from text [108], [109], [110]. Just like RNNs, these networks perform well when millions of labeled examples are present [103]; however, obtaining these large datasets is a non-trivial task. Future approaches that use CNNs or RNNs should consider solutions to obtaining these large quantities of data through means such as weak supervision [111], semi-supervised learning [112] or using pre-trained networks via transfer learning [113], [114].

Semi-supervised learning [112] and weak supervision [111] are techniques that can rapidly construct large datasets for machine learning classifiers. Semi-supervised learning trains classifiers by combining labeled data with unlabeled data. For example, one study used a variational auto encoder with a LSTM network to extract protein–protein interactions from PubMed abstracts and full text [115]. This is an elegant solution for the small dataset problem but requires labeled data to start. This dependency makes finding under-studied relationships difficult as one would need to find or construct examples of the missing relationships at the start.

Weak or distant supervision takes a different approach by using noisy or even erroneous labels to train classifiers [111], [116], [117], [118]. Under this paradigm, sentences are labeled based on their mention pair being present (positive) or absent (negative) in a database and, once labeled, a machine learning classifier can be trained to extract relationships from text [111]. For example, Thomas et al. [119] used distant supervision to train a SVM to extract sentences mentioning protein–protein interactions (PPI). Their SVM model achieved comparable performance against a baseline model; however, the noise generated via distant supervision was difficult to eradicate [119]. A number of efforts have focused on combining distant supervision with other types of labeling strategies to mitigate the negative impacts of noisy knowledge bases [120], [121], [122]. Nicholson et al. [110] found that, in some circumstances, these strategies can be reused across different types of biomedical relationships to learn a heterogeneous knowledge graph in cases where those relationships describe similar physical concepts. Combining distant supervision with other types of labeling strategies remains an active area of investigation with numerous associated challenges and opportunities. Overall, semi-supervised learning and weak supervision provide promising results in terms of relationship extraction and future approaches should consider using these paradigms to train machine learning classifiers.

## 3 Applying knowledge graphs to biomedical challenges

Knowledge graphs can help researchers tackle many biomedical problems such as finding new treatments for existing drugs [9], aiding efforts to diagnose patients [127] and identifying associations between diseases and biomolecules [128]. In many cases, solutions rely on representing knowledge graphs in a low dimensional space, which is a process called representational learning. The goal of this process is to retain and encode the local and/or global structure of a knowledge graph that is relevant to the problem while transforming the graph into a representation that can be readily used with machine learning methods to build predictors. In the following sections we review methods that construct a low dimensional space (Unifying Representational Learning Techniques) and discuss applications that use this space to solve biomedical problems (Unifying Applications).

### 3.1 Unifying representational learning techniques

**Fig. 4:**
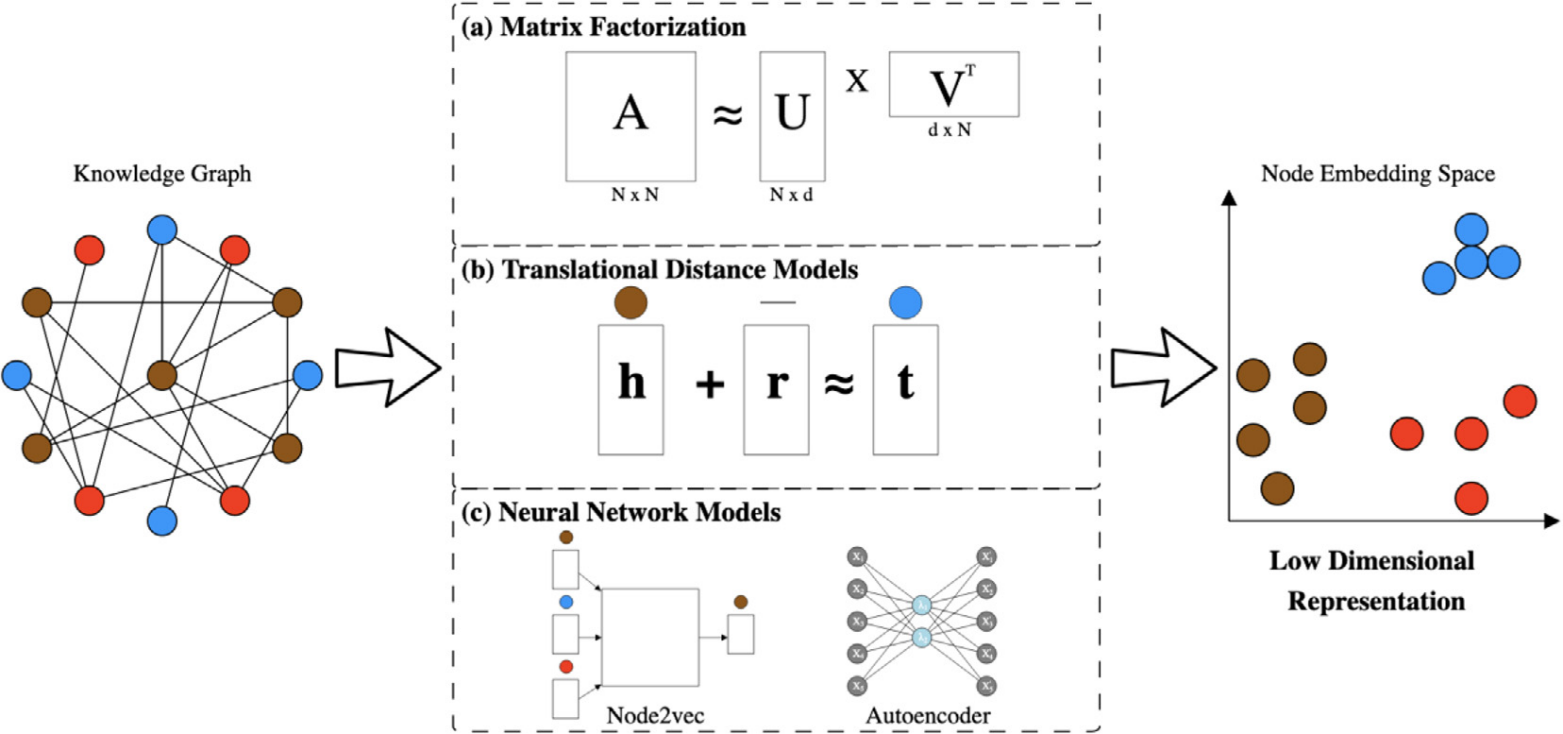
Pipeline for representing knowledge graphs in a low dimensional space. Starting with a knowledge graph, this space can be generated using one of the following options: Matrix Factorization (a), Translational Models (b) or Neural Network Models (c). The output of this pipeline is an embedding space that clusters similar node types together.

Mapping high dimensional data into a low dimensional space greatly improves modeling performance in fields such as natural language processing [104], [105] and image analysis [129]. The success of these approaches served as rationale for a sharper focus on representing knowledge graphs in a low dimensional space [130]. Methods of this class are designed to capture the essence of a knowledge graph in the form of dense vectors [131], [132]. These vectors are often assigned to nodes in a graph [133], but edges can be assigned as well [134]. Techniques that construct a low dimensional space often require information on how nodes are connected with one another [135], [136], [137], [138], while other approaches can work directly with the edges themselves [139]. Once this space has been constructed, machine learning techniques can utilize the space for downstream analyses such as classification or clustering. We group techniques that construct this space into the following three categories: matrix factorization, translational distance models, and neural network models (Fig. 4).

#### 3.1.1 Matrix factorization

Matrix factorization is a class of techniques that use linear algebra to map high dimensional data into a low dimensional space. This projection is accomplished by decomposing a matrix into a set of small rectangular matrices (Fig. 4 (a)). Notable methods for matrix decomposition include Isomap [140], Laplacian eigenmaps [132] and Principal Component Analysis (PCA) [141]/Singular Vector Decomposition (SVD) [131]. These methods were designed to be used on many different types of data; however, we discuss their use in the context of representing knowledge graphs in a low dimensional space and focus particularly on SVD and laplacian eigenmaps.

SVD [131] is an algorithm that uses matrix factorization to portray knowledge graphs in a low dimensional space. The input for this algorithm is an adjacency matrix (A), which is a square matrix where rows and columns represent nodes and each entry is a binary representation of the presence of an edge between two nodes. A is constructed based on the knowledge graph’s structure itself and collapses all edges between two nodes into one unique entity. Following construction, A is decomposed into the following three parts: a square matrix Σ and a set of two small rectangular matrices U and $V^{T}$. Values within Σ are called singular values, which are akin to eigenvalues [131]. Each row in U and each column in $V^{T}$ represents nodes within a low dimensional space [131], [141]. In practice, U is usually used to represent nodes in a knowledge graph and can be used as input for machine learning classifiers to perform tasks such as link prediction or node classification [142]; however, $V^{T}$ has also been used [131], [143]. Typically, matrix factorization algorithms such as SVD are used for recommendation systems via collaborative filtering [144]; however, this technique can also provide a standalone baseline for other relational learning approaches [142].

Laplacian eigenmaps assume there is low dimensional structure in a high dimensional space and preserves this structure when projecting data into a low dimensional space [132]. The first step of this technique is to preserve the low dimensional structure by representing data in the form of a graph where nodes are datapoints and edges are the distance between two points. Knowledge graphs already provide this representation, so no additional processing is necessary at this stage. The second step of this technique is to obtain both an adjacency matrix (A) and a degree matrix (D) from the graph representation. A degree matrix is a diagonal matrix where each entry represents the number of edges connected to a node. The adjacency and degree matrices are converted into a laplacian matrix (L), which is a matrix that shares the same properties as the adjacency matrix. The laplacian matrix is generated by subtracting the adjacency matrix from the degree matrix (L=D-A) and, once constructed, the algorithm uses linear algebra to calculate the laplacian’s eigenvalues and eigenvectors (Lx=λDx). The generated eigenvectors represent the knowledge graph’s nodes represented in a low dimensional space [132]. Other efforts have used variants of this algorithm to construct low dimensional representations of knowledge graphs [135], [136], [145]. Typically, eigenmaps work well when knowledge graphs have a sparse number of edges between nodes but struggle when presented with denser networks [142], [145], [146]. An open area of exploration is to adapt these methods to accommodate knowledge graphs that have a large number of edges.

Matrix factorization is a powerful technique that represents high dimensional data in a low dimensional space. The representation of a knowledge graph in this reduced space does not meet our definition of a knowledge graph; however, this representation supports many use cases including similarity-based (e.g., cosine similarity [147]) and machine learning applications. Common matrix factorization approaches involve using SVD, Laplacian eigenmaps or variants of the two to decompose matrices into smaller rectangular forms. Regarding knowledge graphs, the adjacency matrix (A) is the typical matrix that gets decomposed, but the laplacian matrix (L=D-A) can be used as well. Despite reported success, the dependence on matrices creates an issue of scalability as matrices of large networks may reach memory limitations. Furthermore, the approaches we discussed consider all edge types as equivalent. These limitations could be mitigated by new approaches designed to accommodate multiple node and edge types separately.

#### 3.1.2 Translational distance models

Translational distance models treat edges in a knowledge graph as linear transformations. For example, one such algorithm, TransE [134], treats every node-edge pair as a triplet with head nodes represented as h, edges represented as r, and tail nodes represented as t. These representations are combined into an equation that mimics the iconic word vectors translations (king-man+woman≈queen) from the word2vec model [105]. The described equation is shown as follows: h+r≈t. Starting at the head node (h), one adds the edge vector (r) and the result should be the tail node (t). TransE optimizes vectors for h, r, t, while guaranteeing the global equation (h+r≈t) is satisfied [134]. A caveat to the TransE approach is that it forces relationships to have a one to one mapping, which may not be appropriate for all relationship types.

Wang et al. attempted to resolve the one to one mapping issue by developing the TransH model [148]. TransH treats relations as hyperplanes rather than a regular vector and projects the head (h) and tail (t) nodes onto a hyperplane. Following this projection, a distance vector (${\text{d}}_{r}$) is calculated between the projected head and tail nodes. Finally, each vector is optimized while preserving the global equation: $\text{h}+{\text{d}}_{r}\approx \text{t}$ [148]. Other efforts have built off of the TransE and TransH models [149], [150]. In the future, it may be beneficial for these models to incorporate other types of information such as edge confidence scores, textual information, or edge type information when optimizing these distance models.

#### 3.1.3 Neural networks

Neural networks are a class of machine learning models inspired by the concept of biological neural networks [151]. These networks are reputable for making non-linear transformations of high dimensional data to solve classification and regression problems [151]. In the context of knowledge graphs, the most commonly used structures are based on word2vec [104], [105]. The word2vec term applies to a set of conceptually related approaches that are widely used in the natural language processing field. The goal of word2vec is to project words onto a low dimensional space that preserves their semantic meaning. Strategies for training word2vec models use one of two neural network architectures: skip-gram and continuous bag of words (CBOW). Both models are feed-forward neural networks, but CBOW models are trained to predict a word given its context while skip-gram models are trained to predict the context given a word [104], [105]. Once training is completed, words will be associated with dense vectors that downstream models, such as feed forward networks or recurrent networks, can use for input.

Deepwalk is an early method that represents knowledge graphs in a low dimensional space [152]. The first step of this method is to perform a random walk along a knowledge graph. During the random walk, every generated sequence of nodes is recorded and treated as a sentence in word2vec [104], [105]. After every node has been processed, a skip-gram model is trained to predict the context of each node thereby constructing a low dimensional representation of a knowledge graph [152]. A limitation for deepwalk is that the random walk cannot be controlled, so every node has an equal chance to be reached. Grover and Leskovec demonstrated that this limitation can hurt performance when classifying edges between nodes and developed node2vec as a result [133]. Node2vec operates in the same fashion as deepwalk; however, this algorithm specifies a parameter that lets the random walk be biased when traversing nodes [133]. A caveat to both deepwalk and node2vec is that they ignore information such as edge type and node type. Various approaches have evolved to fix this limitation by incorporating node, edge and even path types when representing knowledge graphs in a low dimensional space [153], [154], [155], [156]. An emerging area of work is to develop approaches that capture both the local and global structure of a graph when constructing this low dimensional space.

Though word2vec is the most common framework used to represent graphs, neural networks are sometimes designed to use the adjacency matrix as input [104], [105]. These approaches use models called autoencoders [157], [158], [159]. Autoencoders are designed to map input into a low dimensional space and then back to a reconstruction of the same input [160], [161]. It is possible to layer on additional objectives by modifying the loss function to take into account criteria above and beyond reconstruction loss [162], [163]. In the context of knowledge graphs, the generated space correlates nodes with dense vectors that capture a graph’s connectivity structure [157], [158], [159]. Despite the high potential of autoencoders, this method relies on an adjacency matrix for input which can run into scalability issues as a knowledge graph asymptotically increases in size [164]. Plus, Khosla et al. discovered that approaches akin to node2vec outperformed algorithms using autoencoders when undergoing link prediction and node classification [164].

Overall, the performance of neural network models largely depends upon the structure of nodes and edges within a knowledge graph [164]. Furthermore, when these approaches are used only nodes are explicitly represented by these vectors. This means a represented knowledge graph no longer meets our definition of a knowledge graph; however, this representation can make it more suitable for many biomedical applications. Future areas of exploration should include hybrid models that use both node2vec and autoencoders to construct complementary low dimensional representations of knowledge graphs.

### 3.2 Unifying applications

**Fig. 5:**
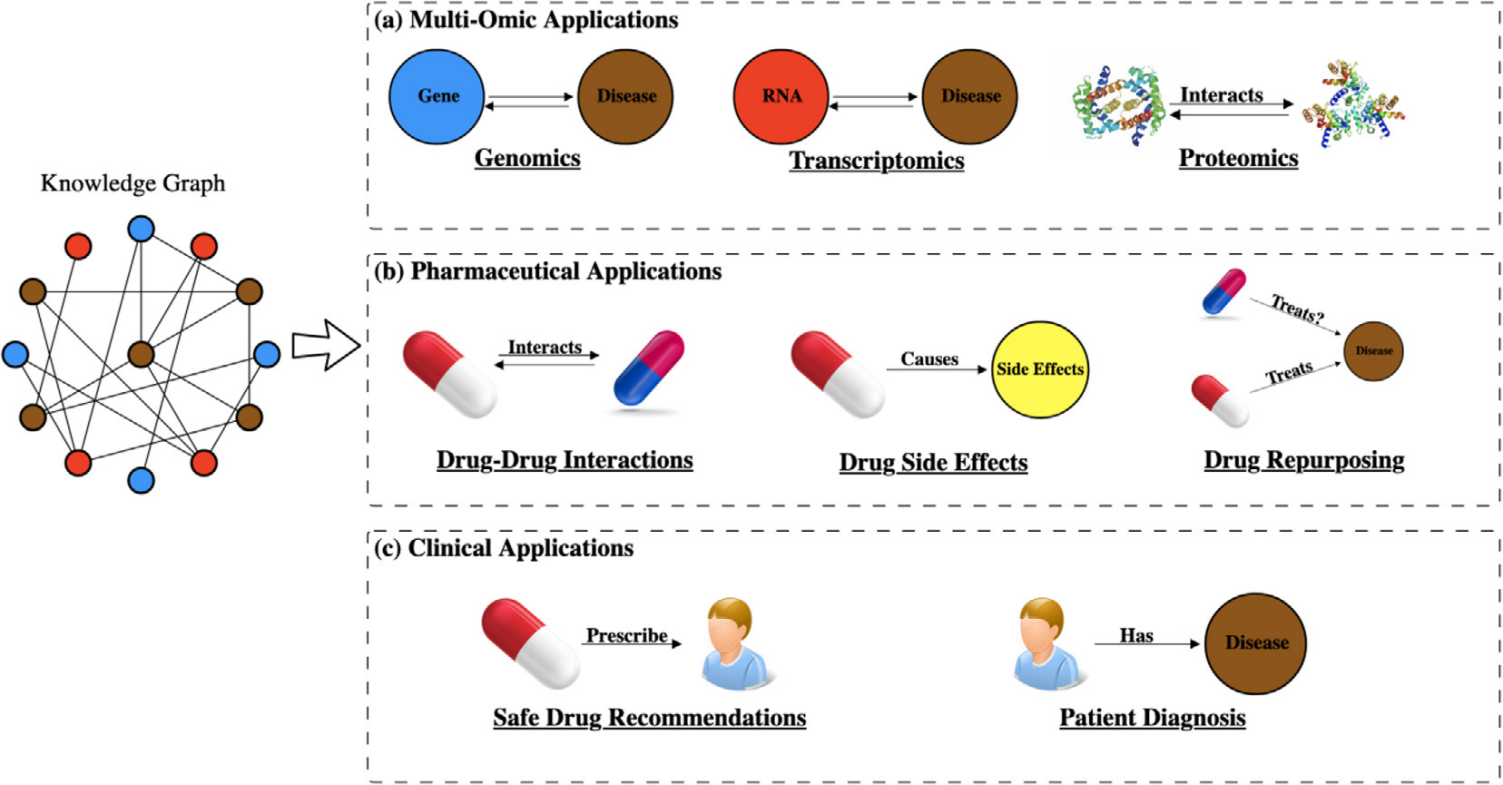
Overview of various biomedical applications that make use of knowledge graphs. Categories consist of: (a) Multi-Omic applications, (b) Pharmaceutical Applications and (c) Clinical Applications.

Knowledge graphs have been applied to many biomedical challenges ranging from identifying proteins’ functions [165] to prioritizing cancer genes [166] to recommending safer drugs for patients [167], [168] (Fig. 5). In this section we review how knowledge graphs are applied in biomedical settings and put particular emphasis on an emerging set of techniques that represent knowledge graphs in a low dimensional space.

#### 3.2.1 Multi-omic applications

Multi-omic applications employ knowledge graphs to study the genome, how genes are expressed in the transcriptome, and how the products of those transcripts interact in the proteome. These graphs are used to establish connections between -omic entities as well as diseases. Tasks in this context include gene-symptom prioritization [169], protein–protein interaction prediction [170], [171] and detecting miRNA-disease associations [128]. We focus specifically on multi-omic applications that represent knowledge graphs in a low dimensional space to make connections.

Recommendation systems make use of knowledge graphs to establish links between RNA with disease and proteins with other proteins. Shen et al. used an algorithm called collaborative filtering to establish an association between miRNA and diseases [128]. The authors constructed a miRNA-Disease network using the Human MicroRNA Disease database (HMDD) [172] and generated an adjacency matrix with the rows representing miRNA and the columns representing diseases. This matrix was decomposed into small rectangular matrices using SVD, then these small matrices were used to calculate similarity scores between miRNAs and diseases. High scores implied a high likelihood that a given miRNA had an association with a given disease [128]. Other approaches built off of Shen et al.’s work by incorporating novel ways to perform matrix factorization [173], [174], [175] or by integrating machine learning models in conjunction with matrix factorization [176]. These approaches achieved high area under the receiver operating curve (AUROC), but new discoveries have been hard to validate as experiments in this space are costly and time consuming at best [128]. Apart from miRNA, collaborative filtering has been used to predict protein–protein interactions [170], [171], [177]. Although extensive validation of newly generated candidates may be impractical, it would be helpful to see future efforts in this space include a blinded literature search for prioritized and randomly selected candidates as part of the standard evaluation pipeline.

Applications of neural network models have mainly used the node2vec model [133] or variants of it. Yang et al. used node2vec to create a recommendation system to infer associations between genes and disease symptoms [169]. The authors constructed a gene-disease symptom knowledge graph by combining two bipartite graphs: genes with diseases and diseases with disease symptoms. The generated graph was embedded via node2vec and similarity scores were calculated for every gene-symptom pair in the graph. High scores implied a high likelihood of an association [169]. This approach outperformed methods that didn’t use a knowledge graph; however, validation was difficult as it involved manual curation of the literature [169]. Similar approaches used variants of node2vec to predict gene-disease associations [8], [178], [179] analyze RNA-seq data [180] and infer novel protein information [165], [181], [182], [183].

Knowledge graphs benefited the multi-omics field as a resource for generating novel discoveries. Most approaches to date use matrix factorization and node2vec to project knowledge graph into a low dimensional space, while translational models (Fig. 4 (b)) may be an untapped resource that could aid future efforts. Another area of exploration could be incorporating multiple sources of information such as compounds, anatomic locations or genetic pathways to improve the specificity of findings (i.e., to predict that a protein–protein interaction happens in a specific cell type or tissue).

#### 3.2.2 Pharmaceutical applications

There are a multitude of examples where knowledge graphs have been applied to identify new properties of drugs. Tasks in this field involve predicting drugs interacting with other drugs [184], identifying molecular targets a drug might interact with [185] and identifying new disease treatments for previously established drugs [186]. In this section we concentrate on applications that apply these graphs to discover new properties of drugs and focus on approaches that use these graphs in a low-dimensional space.

Similar to multi-omic applications, recommendation systems have utilized knowledge graphs to infer novel links between drugs and diseases. Dai et al. used collaborative filtering to infer drug-disease associations [185]. The authors constructed a drug-disease network by integrating two bipartite networks: a drug-gene interaction network and a disease-gene interaction network. They integrated both networks under the assumption that drugs associated with a disease interact with the same gene of interest. Following construction, the authors generated an adjacency matrix where rows represent drugs and columns represent diseases. This matrix was decomposed into two small rectangular matrices and these matrices were used to calculate similarity scores between all drugs and all diseases. High values implied a high chance of an association [185]. Related approaches used this technique to infer drug-target interactions [187], [188], [189] and drug-disease treatments [190], [191], [192], [193], [194]. In spite of reported success, these approaches are limited to the drugs and diseases contained in the graph. Combining these approaches with representations of chemical structures might make it possible to one day make predictions about novel compounds.

Applications that use neural network models have used node2vec [195], [196] and autoencoders [197], [198] approaches to represent knowledge graphs in a low dimensional space. Zong et al. used a node2vec-like model to predict drug-target associations [195]. The authors constructed a disease-target-disease network using drug centered databases: Drugbank [199] and Diseasome [200]. Next, the authors applied a random walk to the graph and trained a skip-gram model to generate a low dimensional representation of the graph. Lastly, the authors constructed a similarity metric that used this space to rank how similar drugs are to their targets [195]. A limitation to this approach is that their graph is missing information such as pharmacological class or drug chemical structure that could improve prediction performance. Overall, neural networks provide a robust set of techniques that have been shown to outperform most linear approaches in this context [201], [202].

Applications that discover new properties of drugs have benefited from using knowledge graphs as a resource. Most methods to date use matrix factorization and neural network models to produce a low-dimensional representation. Due to the success of neural networks [201], [202] much of the field’s focus has shifted to these techniques; however, a possible improvement is to use an ensemble of neural network models and linear methods to improve performance. Another potential avenue for future work would be to incorporate entity-specific hierarchical information or similarity information to improve detection power. For drugs, this could include pharmaceutical classes or chemical structure similarities.

##### 3.2.3 Clinical applications

Clinical applications that use knowledge graphs are in early stages of development, but the long-term goal is to use analyses of these graphs to aid patient care. Typically, graphs for these applications are constructed from electronic health records (EHR): nodes represent patients, drugs and diseases while edges represent relationships such as a patient being prescribed a treatment or a patient being diagnosed with a disease [203], [204], [205], [26]. Tasks within this field range from improving patient diagnoses [206], [207] to recommending safer drugs for patients [167], [207]. We briefly discuss efforts that use knowledge graphs to accomplish such tasks.

Early work in this field applied translational models (Fig. 4 (b)) to knowledge graphs with the goal of recommending safe drugs. Wang et al. used a variant of the TransH [148] model to create such a system for patients [167]. They constructed a disease-patient-drug network by integrating a patient-disease bipartite network with a patient-drug bipartite network. Every node in the newly constructed graph was embedded while satisfying the following equation: h-r≈t. Following the embedding step, the authors formulated their own similarity metric that selected drug combinations with a low number of interactions [167]. Researchers in [150] applied a similar variant of the TransH model to a medical knowledge graph and evaluated their model for link prediction rather than patient recommendation.

In contrast with most applications where node2vec and autoencoder models have become established, this field have focused on using graph attention models [208]. These models mimic machine translation models [209] and aim to simultaneously represent knowledge graphs in a low dimensional space and perform the task at hand. Choi et al. used a graph attention model to predict patient diagnoses [127]. The authors constructed a directed graph using medical concepts from patient EHR data. This directed graph was fed into a graph attention network and then used to predict a patient’s likelihood of heart failure [127]. Other approaches have used graph attention models to perform clinical tasks such as drug safety recommendations [168] and patient diagnoses [210].

Knowledge graphs have shown promising results when used for clinical applications; however, there is still room for improvement. Most approaches have run into the common problem of missing data within EHR [127], [167], [168]. Future directions for the field consist of designing algorithms that can fill in this missing data gap or construct models that can take missing data into account.

## 4 Conclusion

Knowledge graphs are becoming widely used in biomedicine, and we expect their use to continue to grow. At the moment, most are constructed from databases derived from manual curation or from co-occurrences in text. We expect that machine learning approaches will play a key role in quickly deriving new findings from these graphs. Representing these knowledge graphs in a low dimensional space that captures a graph’s local and global structure can enable many downstream machine learning analyses, and methods to capture this structure are an active area of research.

As with any field, rigorous evaluation that can identify key factors that drive success is critical to moving the field forward. In regard to knowledge graphs, evaluation remains difficult. Experiments in this context require a significant amount of time and consequently resources [128], [169]. Moving from open ended and uncontrolled evaluations that consist of describing findings that are consistent with the literature to blinded evaluations of the literature that corroborate predictions and non-predictions would be a valuable first step. There are also well-documented biases related to node degree and degree distribution that must be considered for accurate evaluation [211]. Furthermore, the diversity of applications hinders the development of a standardized set of expected evaluations.

We anticipate that a fruitful avenue of research will be techniques that can produce low dimensional representations of knowledge graphs which distinguish between multiple node and edge types. There are many different sources of bias that lead to spurious edges or incompleteness, and modeling these biases may support better representations of knowledge graphs. It is a promising time for research into the construction and application of knowledge graphs. The peer reviewed literature is growing at an increasing rate and maintaining a complete understanding is becoming increasingly challenging for scientists. One path that scientists can take to maintain awareness is to become hyper-focused on specific areas of knowledge graph literature. If advances in how these graphs are constructed, represented and applied can enable the linking of fields, we may be able to savor the benefits of this detailed knowledge without losing the broader contextual links.

## Funding

David Nicholson Funded by The Gordon and Betty Moore Foundation (GBMF4552) and the National Institutes of Health (T32 HG000046)

Casey S. Greene Funded by The Gordon and Betty Moore Foundation (GBMF4552) and the National Institutes of Health (R01 HG010067).

## CRediT authorship contribution statement

**David** **N.** **Nicholson:** Conceptualization, Funding acquisition, Investigation, Writing - original draft, Visualization. **Casey S. Greene:** Conceptualization, Funding acquisition, Supervision, Writing - review & editing.

## Declaration of Competing Interest

The authors declare that they have no known competing financial interests or personal relationships that could have appeared to influence the work reported in this paper.
